# Integrated genomic analysis identifies novel low-frequency *cis*-regulatory variant rs2279658 associated with VSD risk in Chinese children

**DOI:** 10.3389/fcell.2022.1062403

**Published:** 2022-12-08

**Authors:** Lihui Jin, Zhenyuan Han, Zhongli Jiang, Jieru Lu, Yizhuo Wu, Bingqian Yan, Weibin Zhang, Xuedong Lin, Lvyan Jiang, Pengjun Zhao, Kun Sun

**Affiliations:** ^1^ Department of Pediatric Cardiology, Xinhua Hospital, School of Medicine, Shanghai Jiao Tong University, Shanghai, China; ^2^ Department of Oral and Maxillofacial Surgery, Peking University School and Hospital of Stomatology, Beijing, China; ^3^ Department of Statistics, College of Science, Purdue University, West Lafayette, IN, United States; ^4^ Children’s Heart Center, Institute of Cardiovascular Development and Translational Medicine, The Second Affiliated Hospital and Yuying Children’s Hospital of Wenzhou Medical University, Wenzhou, China; ^5^ Institute for Developmental and Regenerative Cardiovascular Medicine, Xinhua Hospital, School of Medicine, Shanghai Jiao Tong University, Shanghai, China; ^6^ Children’s Hospital of Fudan University, National Children’s Medical Center, Shanghai, China; ^7^ Department of Dermatology, People’s Hospital of Zhengzhou, Zhengzhou, China; ^8^ Department of Gastroenterology, Wenzhou Hospital of Chinese Medicine, Wenzhou, China; ^9^ Ping’an Community Healthcare Center Hospital, Shanghai, China

**Keywords:** VSD, *cis*-regulatory SNP, rs2279658, exome-wide association analysis, *in vitro* cardiac differentiation, *cis*-regulatory region

## Abstract

VSD combined with other cardiac or extracardiac malformations (defined as “complex VSD” by us) is one of the major causes of perinatal morbidity and mortality. Functional non-coding SNPs (*cis*-regulatory SNPs) have not been systematically studied in CHDs, including complex VSD. Here we report an exome-wide association analysis using WES data of 60 PA/VSD cases, 20 TOF cases and 100 controls in Chinese children. We identify 93 low-frequency non-coding SNPs associated with complex VSD risk. A functional genomics pipeline integrating ATAC-seq, ChIP-seq and promoter CHi-C recognizes the rs2279658 variant as a candidate *cis*-regulatory SNP. Specifically, rs2279658 resides in a cardiac-specific enhancer bound by *FOXH1* and *PITX2*, and would abrogate binding of these two transcription factors to the identified enhancer during cardiac morphogenesis. *COQ2* and *FAM175A* are predicted to be target genes for “rs2279658-*FOXH1* or *PITX2*” pairs in the heart. These findings highlight the importance of *cis*-regulatory SNPs in the pathogenesis of complex VSD and broaden our understanding of this disease.

## Introduction

Ventricular septal defect (VSD) is characterized by an interruption in the interventricular septum formation during cardiac morphogenesis, reaching up to 40% of all congenital heart diseases (CHDs) (Penny and Vick, 2011). VSD appears in children as isolated anomalies in most cases, but it can occur in combination with other cardiac or extracardiac defects (hereafter referred to as “complex VSD” defined by us). Tetralogy of Fallot (TOF) and pulmonary atresia with ventricular septal defect (PA/VSD) belonged to complex VSD, and they are one of the leading causes of perinatal morbidity and mortality. Children with complex VSD are at great risk of developing life-threatening complications and need early surgery in clinical practice (Dakkak and Oliver, 2022).

During the past decade, studies in human and animal models have offered insights into the genetic basis for complex VSD. These include typical instances, such as *TBX1* deletion in DiGeorge syndrome (Baldini, 2005), *TBX5* mutations (e.g., c.161T>C, p.Ile54Thr) in Holt-Oram syndrome (Bruneau et al., 2001) and *PTPN11* mutations (e.g., c.923A>G, p.Asn208Ser) in Noonan syndrome (Tartaglia et al., 2002). As research advances, most complex VSD is believed to exhibit multifactorial inheritance attributed to the interaction between low-penetrance variant loci (common variants) and unfavorable environmental elements (Ware and Jefferies, 2012). Current genetic association studies have provided a systematic approach to screen common variants in multiple traits and diseases, including the situation of complex VSD (Dehghan, 2018). However, these studies merely focused on common single nucleotide polymorphisms (SNPs) with a minor allele frequency (MAF) > 0.05 in genomic coding region. Deciphering functional consequences of common non-coding SNPs remains an uncharted territory for complex VSD.

Common non-coding SNPs were reported to account for over 90% of identified disease-associated variants (Maurano et al., 2012). Most of these SNPs were far away from the nearest known gene but enriched in *cis*-regulatory regions. They have been predicted to perturb transcription factor (TF) binding and *cis*-regulatory function to modulate target gene expression, thereby are named *cis*-regulatory SNPs (Gallagher and Chen-Plotkin, 2018). Recently, such “*cis*-regulatory SNP-TF-target gene” circuits have been proven related to inherited risk for cancer and neurodegenerative disorders, which paves the way for our better understanding of *cis*-regulatory SNPs in genetic predisposition to complex VSD (Zhang et al., 2018; Corces et al., 2020).

To identify novel *cis*-regulatory SNPs in complex VSD, we performed an exome-wide association analysis in this study using whole-exome sequencing (WES) data of 80 patients with PA/VSD or TOF and 100 healthy controls. We recognized three common non-coding SNPs in association with complex VSD risk by an additive model in logistic regression analysis. Subsequent functional analysis showed that the SNP rs2279658 resided in an enhancer activated during cardiac differentiation. The risk allele rs2279658-G was further predicted to impair *FOXH1* and/or *PITX2* binding to the identified enhancer, and might cause abnormal transcription of *COQ2* and *FAM175A* in the heart. These results highlight the significance of *cis*-regulatory SNPs in the development of complex VSD and increase our knowledge of this disease.

## Materials and methods

### Study population

Patients and controls included in the exome-wide association analysis have been reported in our previous study (Xie et al., 2019). Briefly, the complete cohort of patients with complex VSD comprised 60 PA/VSD cases and 20 TOF cases, whereas the control cohort consisted of 100 healthy non-hospitalized children. Written informed consents were obtained from all participants or their guardians before enrollment. This study compiled with the Helsinki Declaration and was approved by the Ethics Committee of Xinhua Hospital affiliated to Shanghai Jiao Tong University (No. XHEC-C-2012-018).

### Exome-wide association analysis

WES data of 80 complex VSD cases and 100 controls were derived from our previous study (Xie et al., 2019). No individuals were excluded because of low genotyping rate (<95%) or sex discrepancies. SNPs were excluded if they: 1) were not located on autosomal chromosomes; 2) had a call rate of <95%; 3) had a MAF of <0.05 in controls; 4) had a *p* value of <1 × 10^−5^ in the Hardy-Weinberg equilibrium test among controls. A set of 125,399 SNPs passed quality control criteria and was used for exome-wide association analysis.

Population structure was evaluated by principal component analysis (PCA) as implemented *via* PLINK (version 1.90) (Purcell et al., 2007). The first two eigenvectors were visualized *via* R package “ggplot2”. Exome-wide associations were assessed in an additive model by logistic regression analysis with adjustment for the top eigenvector. SNPs that had a *p* value below 5 × 10^−8^ were considered exome-wide significant.

### Causal SNPs and functional annotation

Results of exome-wide association analysis were functional annotated via FUMA (http://fuma.ctglab.nl/) (Watanabe et al., 2017). Exome-wide significant SNPs that were independent from each other at *r* ^2^ < 0.6 were defined as independent lead SNPs. All filtered SNPs that were in linkage disequilibrium (LD) (*r* ^2^ > 0.2) with one of the independent lead SNPs were included in the LD expansion. Subsequently, causal SNPs were defined by merging independent lead SNPs and corresponding LD SNPs. Functional annotation for these SNPs were acquired *via* ANNOVAR using Ensembl genes (Wang K. et al., 2010).

### Reanalysis of public assay for transposase accessible chromatin with sequencing (ATAC-seq) data

ATAC-seq libraries in human embryonic stem cells (hESCs) were generated by Qing et al. (2019), and in hESC-derived cardiac lineage cells generated by Bertero et al. (2019). Raw fastq files were downloaded from GSE109524 for hESCs, and from GSE106690 for cardiac lineage cells. ATAC-seq reads were trimmed *via* Trim_galore (version 3.4) and mapped to the hg19 genome *via* Bowtie2 (version 2.4.4) with default parameters. Uniquely mapping reads were kept by filtering for their primary mapping location with MAPQ score >25. Duplicate reads were removed *via* Samtools (version 1.9). Significant peaks were called via MACS2 (version 2.1.1.20160309) using the parameters “--nomodel--shift-100 --extsize 200 -q 0.05”. Gene tracks were normalized to reads per genomic content (RPGC) and visualized *via* Integrative Genomics Viewers (IGV).

### Correlation analysis

Genome-wide correlation heatmap was generated using pearson correlation method *via* DeepTools (version 3.4.3) multiBamSummary (Ramirez et al., 2016). Processed bam files were used as inputs. All chromosomes were divided into 10 kb non-overlapping bins in the correlation analysis. The resulting matrix was visualized *via* R package “pheatmap”.

### Read density analysis

Read density values were calculated *via* DeepTools computeMatrix using the parameters “--referencePoint TSS -a 1000 -b 1000”. The resulting matrices were visualized *via* DeepTools plotHeatmap. Of note, read density heatmaps were sorted by mean coverage per locus.

### Reanalysis of public chromatin immunoprecipitation sequencing (ChIP-seq) data

H3K27ac ChIP-seq libraries of normal human heart tissues [specifically, right atrium (RA), left ventricle (LV) and right ventricle (RV)] were generated by the NIH Roadmap Epigenomics Mapping Consortium (Bernstein et al., 2010). Raw fastq files were downloaded from GSE16256. Analysis pipeline for ChIP-seq reads was the same as that for ATAC-seq. Significant narrow peaks were called *via* MACS2 with default parameters. Gene tracks were normalized to RPGC and visualized *via* IGV.

### *In Situ* promoter capture Hi-C (CHi-C)

*In situ* promoter CHi-C libraries in human induced pluripotent stem cells (iPSCs) and iPSC-derived CMs were generated by Montefiori et al. (2018). Processed sequencing data were downloaded from E-MTAB-6014 to find interactions between SNPs and promoters. Selected interactions were visualized *via* IGV.

### Tissue expression analysis

Microarray data of human embryonic heart samples from Carnegie stages 11–15 were obtained from our former study (Shi et al., 2018). Gene expression data of human adult hearts were retrieved from the Genotype-Tissue Expression (GTEx) database (GTEx Consortium, 2020).

## Results

### Integrated analysis framework for “*cis*-regulatory SNP-TF-target gene” characterization

**FIGURE 1:**
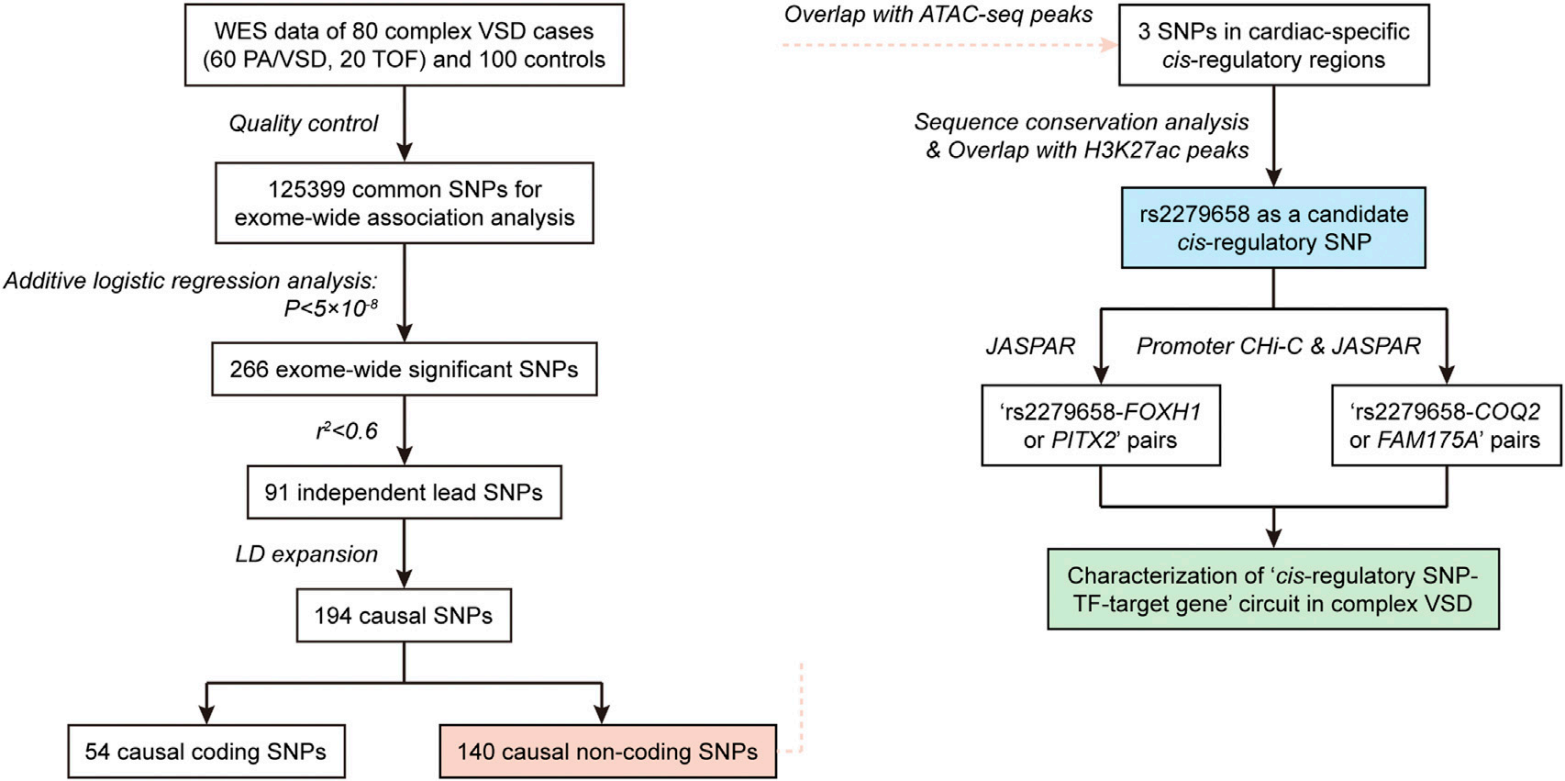
Flow chart showing the integrated pipeline used to recognize triplets of *cis*-regulatory SNP, corresponding TF and target gene in complex VSD.

We proposed an integrated multi-omic analysis to systematically recognize triplets of *cis*-regulatory SNP, relevant TF and target gene in complex VSD. Our hypothesis was that *cis*-regulatory SNPs modulating the risk of complex VSD would probably reside in *cis*-regulatory regions to disrupt the binding affinity of corresponding TFs, which then could alter transcription activity of their target genes in the heart. The schematic illustration of our framework was shown in Figure 1. Briefly, “*cis*-regulatory SNP-TF” pairs were identified by combining exome-wide association analysis, epigenetic annotation of *cis*-regulatory regions and motif analysis. Putative target genes were mainly determined based on promoter CHi-C analysis and binding site prediction.

### Identifying causal non-coding SNPs *via* exome-wide association analysis

**FIGURE 2:**
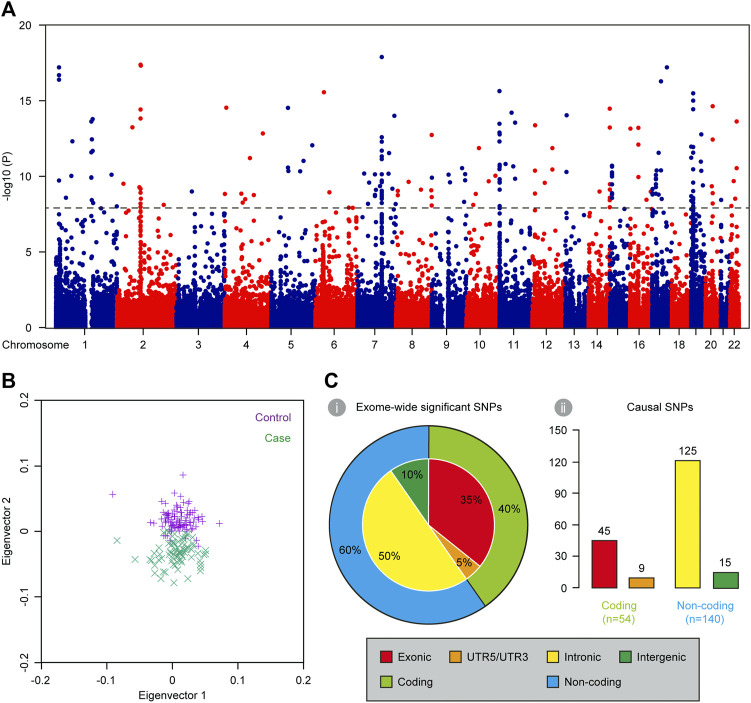
Characterization of causal SNPs for complex VSD *via* exome-wide association analysis. **(A)** Manhattan plot showing the base-pair position of each SNP along autosomal chromosomes on the *x* axis and −log_10_-transformed *p* values on the *y* axis (*n* = 180). The black dashed line indicates the threshold for exome-wide significance (*p* < 5 × 10^–8^). **(B)** PCA plot showing the first two eigenvectors of 80 complex VSD cases and 100 controls. **(C)** Genomic distribution of exome-wide significant SNPs (i) and causal SNPs (ii).

Population structure of the 80 complex VSD cases and 100 controls was described in PCA plot (Figure 2B). There was remarkable population stratification between cases and controls, but little heterogeneity between PA/VSD and TOF individuals. To reduce false associations induced by population stratification, we assessed the exome-wide association in an additive logistic regression model with adjustment for the top eigenvector. The overall *p* values were presented in Figure 2A and 266 SNPs exhibited a significant association with *p* < 5 × 10^−8^ (Supplementary Table S1). Of all the exome-wide significant SNPs, 95 (35%) were in exonic regions, 133 (50%) were in intronic regions, 12 (5%) were in UTR5/UTR3 regions, and 26 (10%) were in intergenic regions (Figure 2C). We used FUMA to functionally annotate results from the association analysis and extracted 91 independent lead SNPs. Noteworthy, most independent lead SNPs in the locus were not necessarily the causal SNP but that it might be in LD with the causal one (Bush and Moore, 2012). Following the criteria of *r* ^2^ > 0.2, we examined every 2-Mb region centered on each lead SNP and identified 143 LD SNPs. Together, our exome-wide association analysis obtained a total of 194 causal SNPs (Supplementary Table S2). Of all the causal SNPs, 140 (72%) were in non-coding regions, and thereby were included in subsequent functional analysis for *cis*-regulatory SNP characterization (Figure 2C).

### Profiling of *cis*-regulatory regions during cardiac morphogenesis

**FIGURE 3:**
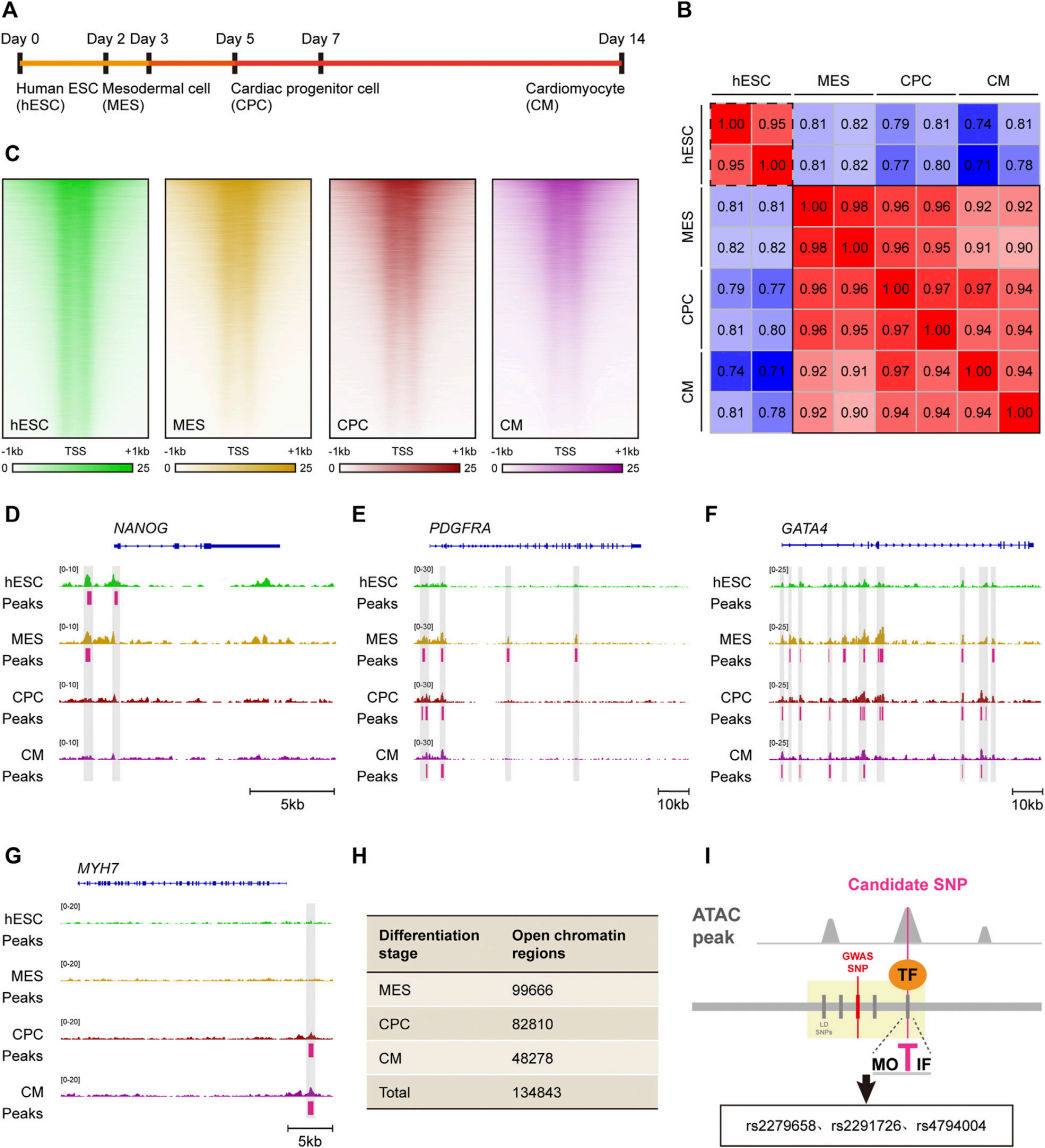
Identification of candidate *cis*-regulatory SNPs based on *in vitro* cardiac differentiation model. **(A)** Schematic of *in vitro* cardiac differentiation of hESCs. **(B)** Correlation heatmap of ATAC-seq accessibility in hESC, MES, CPC and CM. Samples with similar chromatin accessibility are highlighted by black solid or dashed border. **(C)** Read density heatmaps showing ATAC-seq signal enrichment at 41,029 genomic regions in hESC, MES, CPC, and CM. Each signal is represented as a horizontal line, compared in a 2 kb summit-centered window, and ranked vertically by signal strength. **(D–G)** IGV views of the *NANOG* **(D)**, *PDGFRA* **(E)**, *GATA4* **(F)** and *MYH7* **(G)** locus. ATAC-seq peaks activated in hESC, MES, CPC or CM are highlighted in gray. **(H)** Number of open chromatin regions in MES, CPC and CM as well as total open chromatin regions during the entire cardiac differentiation process. **(I)** Visual illustration of the analytical pipeline. Candidate *cis*-regulatory SNPs are recognized among causal non-coding SNPs by overlapping with ATAC-seq peaks.

Cardiac morphogenesis is a tightly-regulated process involving multiple stages and perturbations in any stage can lead to complex VSD. *In vitro* directed differentiation of hESC into CM serves as an ideal system to elucidate spatiotemporal regulation of cardiogenesis (Murry and Keller, 2008). As chromatin accessibility was a hallmark of genomic regulatory regions, we analyzed public ATAC-seq datasets for hESC, mesodermal cell (MES), cardiac progenitor cell (CPC) and CM to obtain cardiac-specific *cis*-regulatory regions (Figure 3A). Interestingly, chromatin accessibility patterns of MES, CPC, and CM were extremely similar as revealed by ATAC-seq, but these patterns were far different from that of hESC (Figures 3B,C), indicating a strong conservation of *cis*-regulatory regions within cardiac lineage cells. Considering the previous report that such regulatory regions were cell-type specific in brain (Nott et al., 2019), we next asked whether our finding reflected the nature of cardiac morphogenesis. A high-resolution examination of ATAC-seq enrichment near lineage specification genes was conducted in all *in vitro* cardiogenic stages, which suggested a high correlation between chromatin accessibility and transcriptional activity. Notable examples included *NANOG* in the hESC, *PDGFRA* in the MES, *GATA4* in the CPC and *MYH7* in the CM (Figures 3D–G). Significant ATAC-seq peaks were observed at promoters or putative enhancers at the same stages when they were expressed. Thus, owing to the similar cardiogenic expression pattern in distinct cardiac lineage cells, it is convincing that chromatin accessibility and *cis*-regulatory regions remain little changed during cardiac differentiation process.

According to the retrieved ATAC-seq datasets, there were 99,666 open chromatin regions in MES, 82,810 in CPC as well as 48,278 in CM (Figure 3H). A total of 134,843 open chromatin regions were identified during cardiac morphogenesis after merging those in MES, CPC, and CM, and were recognized as cardiac-specific *cis*-regulatory regions.

### rs2279658 as the candidate *cis*-regulatory SNP for complex VSD

**FIGURE 4:**
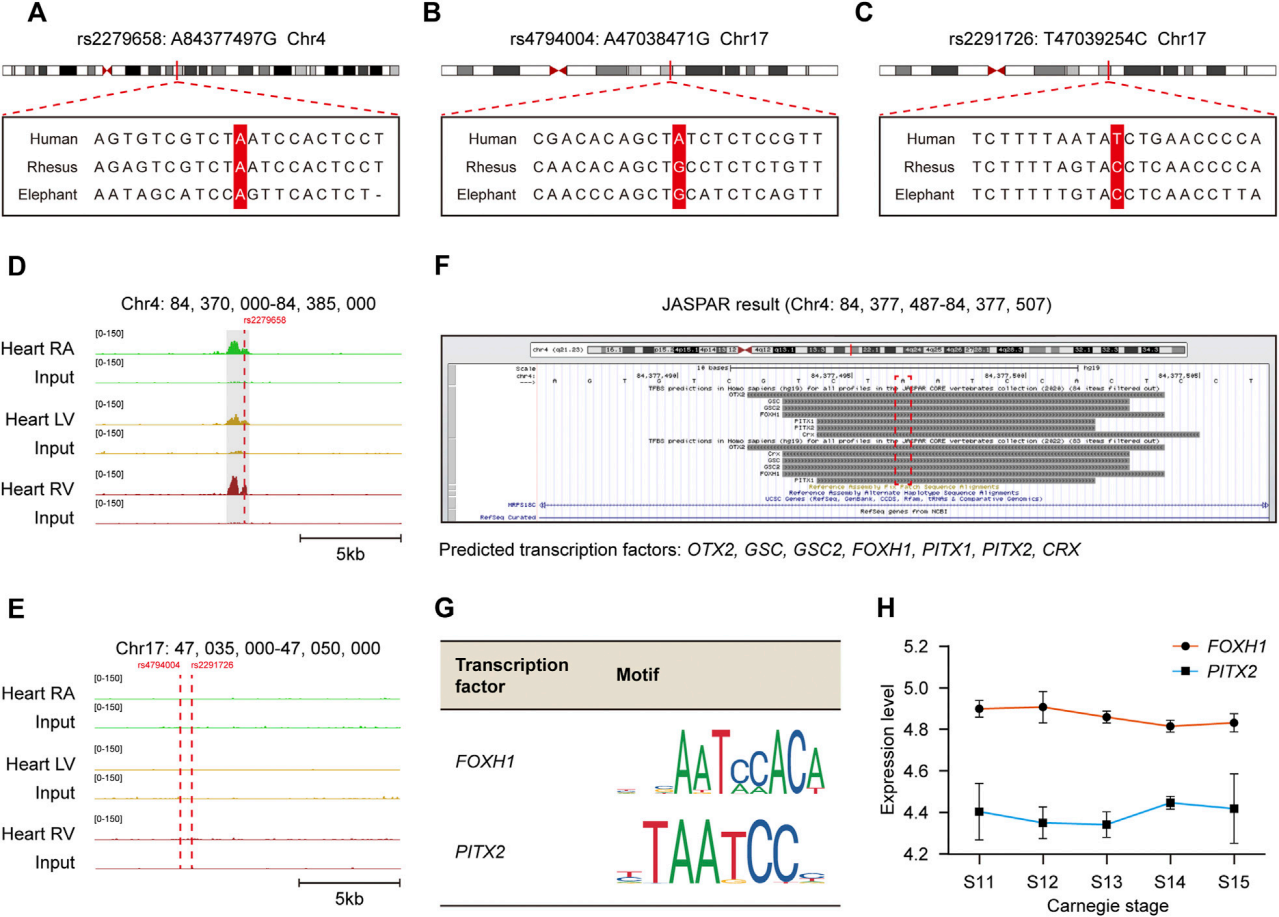
Recognization of *cis*-regulatory SNP rs2279658 related to complex VSD susceptibility and its corresponding TFs. **(A–C)** Alignment of the human, rhesus and elephant DNA sequences in loci around SNP rs2279658 **(A)**, rs4794004 **(B)** and rs2291726 **(C)**, respectively. The SNP loci are highlighted in red. **(D–E)** IGV views of genomic loci around SNP rs2279658 **(D)**, rs4794004 **(E)** and rs2291726 **(E)**. H3K27ac peaks activated in hESC, MES, CPC or CM are highlighted in gray. **(F)** Predicted TFs bound to the rs2279658 locus *via* JASPAR database. **(G)** Motifs of *FOXH1* and *PITX2*. **(H)** Line plot showing expression levels of *FOXH1* and *PITX2* across human cardiac morphogenesis.

The schematic representation of identifying *cis*-regulatory SNP candidates for complex VSD was shown in Figure 3I. We found three of 140 causal non-coding SNPs resided in cardiac-specific *cis*-regulatory regions, namely rs2279658, rs4794004, and rs2291726 (Figure 3I). Of note, the SNP rs2279658 was located at the center of a short sequence conserved among humans, rhesus monkeys and elephants (Figure 4A). On the contrary, genomic sequences containing rs4794004-A or rs2291726-T varied among the aforementioned species (Figures 4B,C). Given that these three SNPs lay in intergenic regions, we then asked whether their corresponding *cis*-regulatory regions had enhancer activity in the heart. Histone acetylation such as H3K27ac has been well recognized as a marker for active enhancers and an excellent indicator of enhancer activity (Creyghton et al., 2010). Thus, we retrieved H3K27ac ChIP-seq datasets produced by the NIH Roadmap Epigenomics Mapping Consortium from normal human heart tissues of left atrium as well as left and right ventricle (Bernstein et al., 2010). There was significant H3K27ac enrichment at the rs2279658-A locus in all tissues examined, whereas the rest two exhibited undetectable H3K27ac signals (Figures 4D,E). With these above evidences we inferred rs2279658 to be an important *cis*-regulatory candidate associated with complex VSD.

To predict TFs potentially affected by *cis*-regulatory SNP rs2279658, we profiled direct interactions between the rs2279658-A containing sequence and TFs *via* JASPAR database (Castro-Mondragon et al., 2022). Several well-studied TFs were found to recognize and bind to the selected sequence, including *CRX*, *FOXH1*, *GSC*, *GSC2*, *OTX2*, *PITX1*, and *PITX2* (Figure 4F). Among these TF candidates, *FOXH1* and *PITX2* have been formerly reported to play critical roles in embryonic heart development and thereby triggered our interest. Motif analysis revealed that both *FOXH1* and *PITX2* recognized a 5-bp consensus sequence “AATCC” to promote or inhibit the assembly of transcription machinery (Figure 4G), so that the risk allele rs2279658-G would abrogate binding of these 2 TFs to corresponding *cis*-regulatory regions. Besides, considerable mRNA levels of *FOXH1* and *PITX2* were observed in human embryonic heart from Carnegie stage 11–15 (Figure 4H), despite few in human adult heart (Supplementary Figures S1A,B). In general, the risk allele rs2279658-G can indeed be associated with complex VSD in a *cis*-regulatory manner.

### Determining target genes regulated by “rs2279658-TF” pairs

**FIGURE 5:**
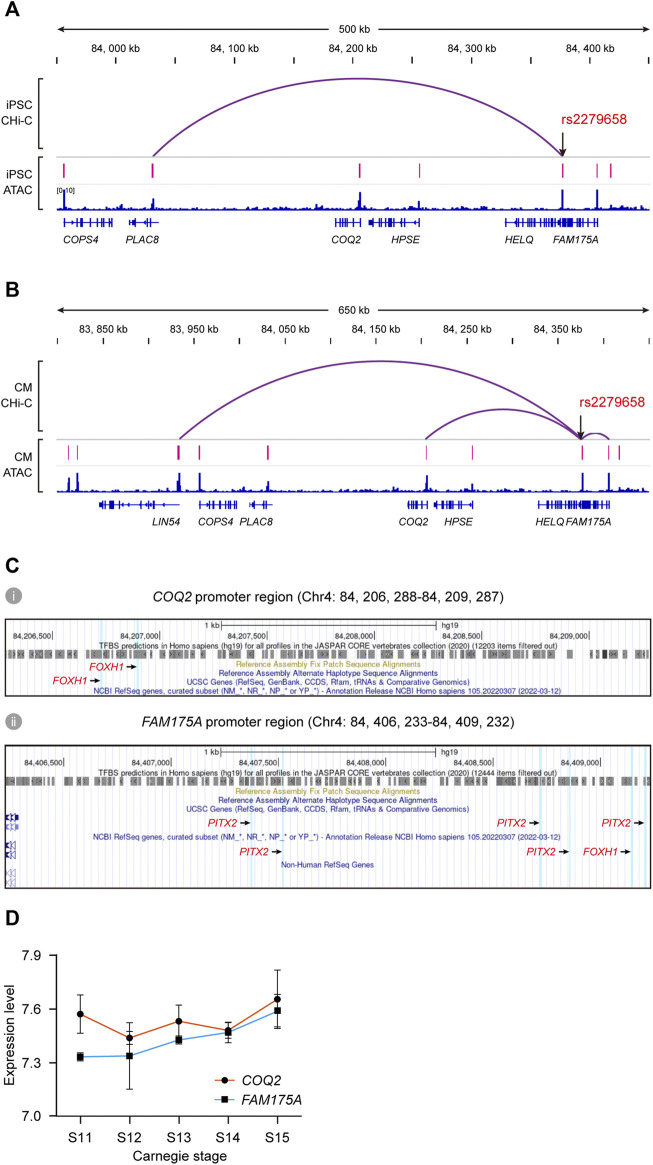
Identification of potential target genes of *cis*-regulatory SNP rs2279658. **(A,B)** IGV views of significant promoter CHi-C loops at the rs2279658 locus in iPSC **(A)** and CM **(B)**. **(C)** Predicted TF binding sites in promoter regions of *COQ2* (i) and *FAM175A* (ii), respectively. *FOXH1-* and *PITX2-*binding sites are highlighted in blue. **(D)** Line plot showing expression levels of *COQ2* and *FAM175A* across human cardiac morphogenesis.

We explored a public promoter interaction data for cardiovascular disease genetics to pinpoint target genes regulated by rs2279658. The chromatin interaction map, developed *via in situ* promoter CHi-C recorded high-resolution promoter-enhancer interactions of human iPSCs and iPSC-derived CMs (Montefiori et al., 2018). rs2279658 was found to link to the *PLAC8* gene promoter in iPSCs (*p* < 0.05) (Figure 5A), and to *LIN54*, *COQ2*, and *FAM175A* gene in CMs (*p* < 0.05) (Figure 5B). This data accorded with the above-mentioned notion that *cis*-regulatory regions varied among cell types, so that such promoter-enhancer interactome was applicable for profiling “*cis*-regulatory SNP-target gene” pairs in complex VSD. Since the CM promoter interaction map linked SNPs to target genes relevant with cardiovascular disease, *LIN54*, *COQ2*, and *FAM175A* were prioritized for further TF correlation analysis.

According to the JASPAR database, there existed two binding sites of *FOXH1* in the *COQ2* gene promoter (Figure 5C), as well as binding sites of both *FOXH1* and *PITX2* in the *FAM175A* promoter (Figure 5C). By contrast, the binding site of neither *FOXH1* nor *PITX2* was predicted in the *LIN54* gene promoter (data not shown). Tissue expression analysis showed that expression of *COQ2* and *FAM175A* could be detected in fetal heart from Carnegie stage 11–15 (Figure 5D) as well as human adult heart (Supplementary Figures S1C,D). The risk allele rs2279658-G was also related to decreased expression of *FAM175A* in the whole blood (data not shown) (Jansen et al., 2017). Taken together, we conjectured that the rs2279658 A>G change might affect complex VSD risk by changing the level of *COQ2* or *FAM175A*.

## Discussion

This study presented a computational pipeline for systematically examining the functional readouts of causal noncoding SNPs. We applied this method to our previously published WES data of complex VSD, and discovered a low-frequency noncoding SNP rs2279658 with multi-omic evidence supporting its role in modulating binding affinities of *FOXH1* and/or *PITX2*. Generally, rs2279658 was predicted to exert its function through TF-mediated transcription regulation, so that it was recognized as a *cis*-regulatory SNP related to complex VSD risk. *Cis*-regulatory SNPs garner substantial research interest in recent years as an emerging category of disease-relevant inherited variation. They have been deciphered in many complex genetic diseases *via* epigenetic profile. For example, the risk allele rs4321755-T identified in a breast cancer susceptibility region could create a *GATA3*-binding motif within an enhancer, thereby leading to altered *GATA3* binding and chromatin accessibility in breast cancer (Zhang et al., 2018). Another study carried out by M. Ryan and colleagues provided a high-resolution epigenetic profile of the functional consequences of inherited regulatory SNPs in Alzheimer’s and Parkinson’s diseases (Corces et al., 2020). These reports provide an avenue towards the nomination of novel therapeutic targets that formerly remained neglected due to the complexity of dissecting the noncoding genome. Similar studies are rare in CHDs and our finding of the novel *cis*-regulatory SNP in complex VSD will foster progress of this field.

In addition, the multi-omic method facilitated the recognition of putative target genes by combining promoter CHi-C and tissue expression analysis. In a promoter CHi-C map from iPSC-derived CMs, significant interactions were found between the rs2279658-A containing sequence and promoters of *COQ2* and *FAM175A*. As promoter CHi-C is capable to precisely map distal genomic regions interacted with a promoter, this approach can help reduce the false discovery rate that currently challenge when predicting target genes (Montefiori et al., 2018). Factually, the rs2279658-A locus is ∼175 kb away from the *COQ2* promoter and ∼40 kb from the *FAM175A* promoter, which meets the conventional notion that *cis*-regulatory regions can be located kilobases from their target genes (Smemo et al., 2014). *COQ2* encodes a well-studied homolog of mitochondrial coenzyme Q oxidoreductases essential for mitochondrial function and is linked to the susceptibility of coronary heart disease (Gharib et al., 2012). *FAM175A* encodes a protein that binds to the C-terminal repeats of BRCA1 and is involved in cell cycle checkpoint control (Draga et al., 2015). The role of *COQ2* and *FAM175A* in the development of VSD remains obscure. Nonetheless, given the baseline mRNA level of *FAM175A* and *COQ2* in human embryo hearts, they are likely to contribute to complex VSD predisposition and requires further investigation.

**FIGURE 6:**
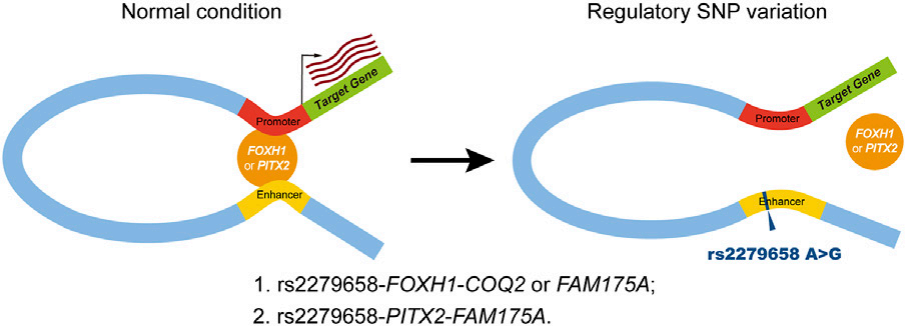
Schematic of the putative working model for *cis*-regulatory SNP rs2279658 in complex VSD pathogenesis. Left panel (normal condition), the cardiac-specific enhancer carrying the protective allele rs2279658-A has known motifs for *FOXH1* and *PITX2*, resulting in high-affinity binding of *FOXH1* and/or *PITX2*, which makes this enhancer active in regulating its target genes via chromatin looping. *Right panel* (regulatory SNP variation), the cardiac-specific enhancer harboring the risk allele rs2279658-G has disrupted *FOXH1*- and *PITX2*-binding motifs, thereby weakening the interaction between this enhancer and promoters of target genes.

Our comprehensive analysis characterized candidate triplets of *cis*-regulatory SNP, corresponding TF and target genes in complex VSD, which addressed our hypothesis that *cis*-regulatory SNPs might affect TF-binding activities. To avoid false positives, we integrated cell type-specific epigenetic profiles, motif analysis and binding site prediction in this study. Our method found that the risk allele rs2279658-G would destroy the binding of two CHD-associated TFs, *FOXH1* and *PITX2*, within a cardiac-specific enhancer. *FOXH1* belongs to the forkhead/winged-helix family whose mutation (c.659_660ins.C) has been reported in Chinese VSD patients (Wang B. B. et al., 2010). *PITX2* is a well-established regulators of cardiac left-right asymmetry and CHD including VSD (Franco et al., 2017). On the other hand, binding sites of *FOXH1* and/or *PITX2* were identified in the promoter of putative target genes *COQ2* and *FAM175A*. These results conformed to a widely accepted model of enhancer-driven gene regulation via chromatin looping (Figure 6) (de Laat et al., 2008). In eukaryotes, the enhancer and its interacting promoters within a topologically associating domain form “hairpin loops” to facilitate enhancer/promoter interactions. The “hairpin loop” brings TFs bound to the enhancer to TFs bound to RNA polymerase II on the promoter, resulting in subsequent transcript activation.

In conclusion, our integrative computational pipeline has identified a novel *cis-*regulatory SNP that may modulate the predisposition to complex VSD. This study is limited by a small size of VSD cases and controls in the exome-wide association analysis, as well as the lack of evidence from mice and cell lines. Nonetheless, our method can be extended to study *cis*-regulatory SNPs modulating susceptibility of other CHDs, contributing to understanding new pathways in cardiac morphogenesis and CHD development.

## Data Availability

The datasets presented in this study can be found in online repositories. The names of the repository/repositories and accession number(s) can be found in the article/Supplementary Material.
