# Supplementary figures and images for: Integrated genomic analysis identifies novel low-frequency cis-regulatory variant rs2279658 associated with VSD risk in Chinese children
Source: Front Cell Dev Biol. 2022 Dec 8;10:1062403. doi: 10.3389/fcell.2022.1062403 (PMC9773552; doi:10.3389/fcell.2022.1062403)

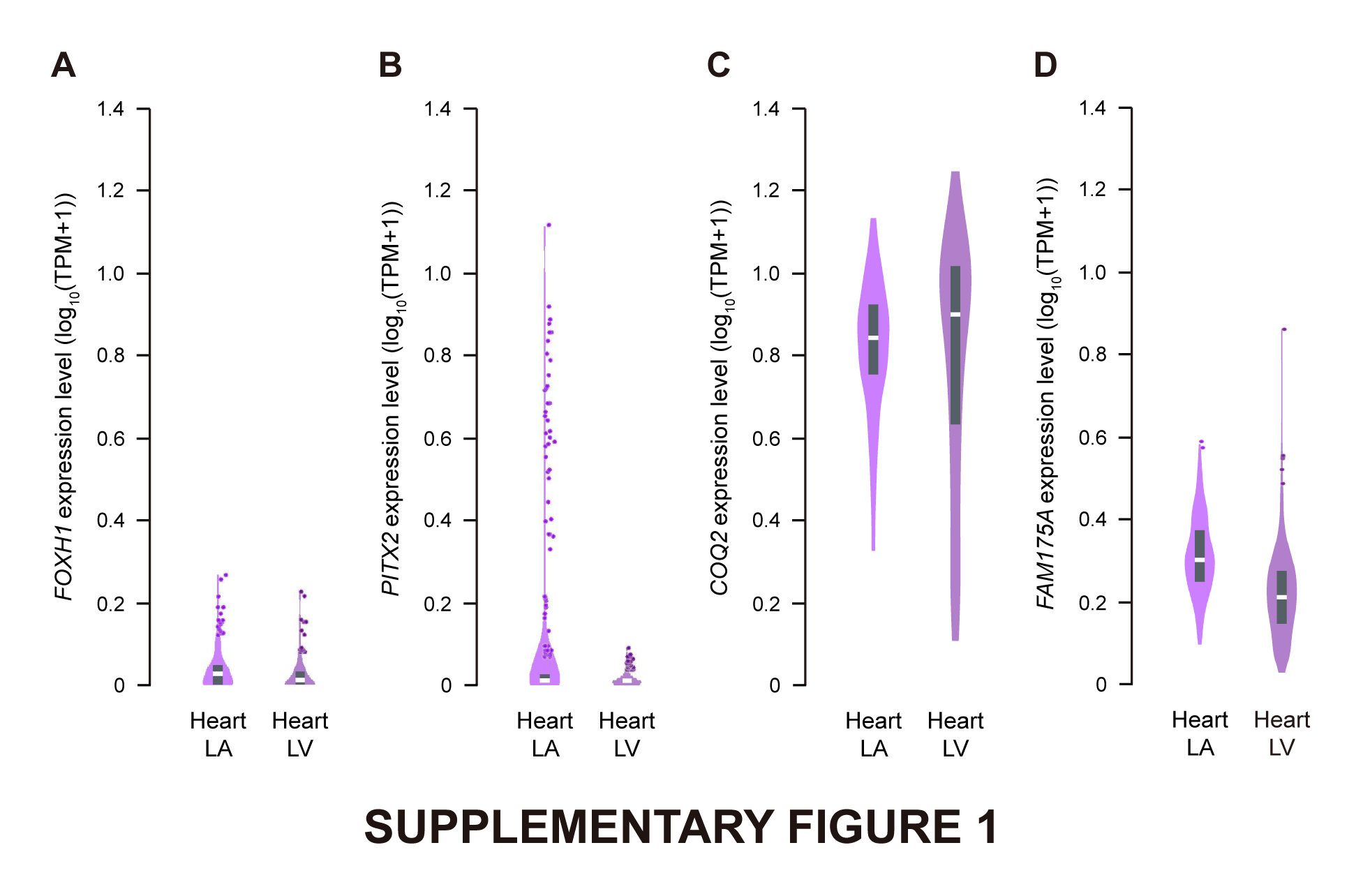

Supplement: Supplementary file 2 [file Image1.TIF]
