# Supplementary material for: Integrated genomic analysis identifies novel low-frequency cis-regulatory variant rs2279658 associated with VSD risk in Chinese children
Source: Front Cell Dev Biol. 2022 Dec 8;10:1062403. doi: 10.3389/fcell.2022.1062403 (PMC9773552; doi:10.3389/fcell.2022.1062403)
Supplement: Supplementary file 3 [file DataSheet1.pdf]

Table 1 | Accession of public sequencing data used in the study

Sequencing type	Samples	Reference	Accession
Whole exome sequencing	PA/VSD cases	Xie et al.	/
	TOF cases		
	Healthy controls		
Microarray	Human embryonic heart tissues	Shi et al.	/
ATAC-seq	hESC	Li et al.	GSE109524
	MES	Bertero et al.	GSE106690
	CPC		
	CM		
H3K27ac ChIP-seq	Human heart tissues	Bernstein et al.	GSE16256
Promoter CHi-C	Human iPSC	Montefiori et al.	E-MTAB-6014
	iPSC-derived CM		

Reference

Bernstein, B. E., Stamatoyannopoulos, J. A., Costello, J. F., Ren, B., Milosavljevic, A., Meissner, A., et al. (2010). The NIH Roadmap Epigenomics Mapping Consortium. *Nat. Biotechnol.* 28(10), 1045-1048. doi: 10.1038/nbt1010-1045

Bertero, A., Fields, P. A., Ramani, V., Bonora, G., Yardimci, G. G., Reinecke, H., et al. (2019). Dynamics of genome reorganization during human cardiogenesis reveal an RBM20-dependent splicing factory. *Nat. Commun.* 10(1), 1538. doi: 10.1038/s41467-019-09483-5

Li, Q. V., Dixon, G., Verma, N., Rosen, B. P., Gordillo, M., Luo, R., et al. (2019). Genome-scale screens identify JNK-JUN signaling as a barrier for pluripotency exit and endoderm differentiation. *Nat. Genet.* 51(6), 999-1010. doi: 10.1038/s41588-019-0408-9

Montefiori, L. E., Sobreira, D. R., Sakabe, N. J., Aneas, I., Joslin, A. C., Hansen, G. T., et al. (2018). A promoter interaction map for cardiovascular disease genetics. *Elife* 7, e35788. doi: 10.7554/eLife.35788

Shi, X., Huang, T., Wang, J., Liang, Y., Gu, C., Xu, Y., et al. (2018). Next-generation sequencing identifies novel genes with rare variants in total anomalous pulmonary venous connection. *EBioMedicine* 38, 217-227. doi: 10.1016/j.ebiom.2018.11.008

Xie, H., Hong, N., Zhang, E., Li, F., Sun, K., and Yu, Y. (2019). Identification of Rare Copy Number Variants Associated With Pulmonary Atresia With Ventricular Septal Defect. *Front. Genet.* 10, 15. doi: 10.3389/fgene.2019.00015
