# Supplementary material for: Integrated genomic analysis identifies novel low-frequency cis-regulatory variant rs2279658 associated with VSD risk in Chinese children
Source: Front Cell Dev Biol. 2022 Dec 8;10:1062403. doi: 10.3389/fcell.2022.1062403 (PMC9773552; doi:10.3389/fcell.2022.1062403)
Supplement: Supplementary file 4 [file Table3.DOCX]

**Integrated Genomic Analysis Identifies Novel Low-frequency *Cis*-regulatory Variant rs2279658 Associated with VSD Risk in Chinese Children**

Lihui Jin^†^, Zhenyuan Han^†^, Zhongli Jiang^†^, Jieru Lu, Bingqian Yan, Yizhuo Wu, Weibin Zhang,

Xuedong Lin, Lvyan Jiang, Pengjun Zhao*, Kun Sun*

**Supplementary Information**

**Supplementary Figure 1**

**Supplementary Tables 1-2**

**FIGURE LEGEND**

**SUPPLEMENTARY FIGURE 1 |** Expression levels of *FOXH1*, *PITX2*, *COQ2* and *FAM175A* in human adult heart. **(A-D)** Violin plots showing expression levels of *FOXH1* (A), *PITX2* (B), *COQ2* (C) and *FAM175A* (D) in the human LV and LA, respectively.
